# Reply: Issues regarding graft selection and surgical strategy

**DOI:** 10.1016/j.xjon.2025.09.037

**Published:** 2025-10-08

**Authors:** Yuki Kuroda, Hiroki Shiomi, Kenji Minatoya

**Affiliations:** a Department of Cardiovascular Surgery, Kyoto University Graduate School of Medicine, Kyoto, Japan; b Department of Cardiovascular Medicine, Kyoto University Graduate School of Medicine, Kyoto, Japan

Reply to the Editor:

**Figure:**
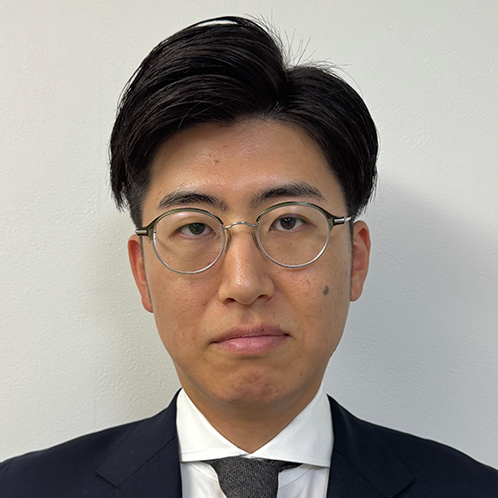

In our recent analysis of the impact of low body mass index (BMI) on clinical outcomes after coronary artery bypass graft (CABG) surgery using the CREDO-Kyoto (Coronary Revascularization Demonstrating Outcome Study in Kyoto) Percutaneous Coronary Intervention/CABG registries, we concluded that having underweight was associated with increased short- and long-term mortality after CABG, especially in men, whereas having overweight or obesity was associated with decreased long-term mortality after CABG.^1^

In response to the publication of our work, Dr Narayan wrote a letter, titled: “Interpreting Low Body Mass Index Outcomes After Coronary Artery Bypass Grafting: The Need for Mechanistic Data and Stratified Analyses.”^2^ First, as he pointed out, the use of lower internal thoracic artery in patients with a low BMI may have predisposed these patients to early graft failure and postoperative myocardial infarction. However, the incidences of myocardial infarction beyond 30 days after surgery and repeat revascularization within and beyond 30 days after surgery were similar. Therefore, although data on graft selection rationale were lacking, we believe the impact of graft selection on our results was limited.

Second, he indicated that within the low-BMI group, a comparison of outcomes by surgical technique (on-pump vs off-pump) could have provided valuable insights, especially regarding perioperative myocardial infarction, stroke, and early cardiovascular mortality. In the low-BMI group, the cumulative 30-day incidences of cardiovascular death, myocardial infarction, and stroke after CABG were numerically lower in patients with off-pump CABG than in patients with on-pump CABG (off-pump: 1.8%, and on-pump: 4.7%, log-rank *P* = .15 for cardiovascular death; off-pump: 1.2%, and on-pump: 2.7%, log-rank *P* = .35 for stroke; and off-pump: 3.0%, and on-pump: 5.9%, log-rank *P* = .21 for myocardial infarction). Considering the limited statistical power for subgroup analyses in our study, further research would be warranted regarding optimal surgical strategies in patients with low BMI.

Finally, he suggested an additional analysis on the basis of data regarding sarcopenia, nutritional biomarkers, or more granular cause-of-death information. Unfortunately, our data lacked such detailed information, and we were unable to show that noncardiovascular deaths in the low-BMI group increased as a result of sarcopenia or malnutrition. Further studies would be needed to address this issue. We thank Dr Narayan for his efforts to strengthen the clinical implications of our study.

## Conflict of Interest Statement

The authors reported no conflicts of interest.

The *Journal* policy requires editors and reviewers to disclose conflicts of interest and to decline handling or reviewing manuscripts for which they may have a conflict of interest. The editors and reviewers of this article have no conflicts of interest.
